# Retrospective study of biopsied head and neck lesions in a cohort of referral Taiwanese patients

**DOI:** 10.1186/1746-160X-10-28

**Published:** 2014-07-21

**Authors:** Frank Lei, Ping-Ho Chen, Jing-Yi Chen, Wen-Chen Wang, Li-Min Lin, Hsien-Cheng Huang, Kun-Yen Ho, Chung-Ho Chen, Yuk-Kwan Chen

**Affiliations:** 1 Division of Periodontology, Kaohsiung Medical University Hospital, Kaohsiung, Taiwan; 2 School of Dentistry, College of Dental Medicine, Kaohsiung Medical University, Kaohsiung, Taiwan; 3 Division of Oral Pathology & Maxillofacial Radiology, Kaohsiung Medical University Hospital, Kaohsiung, Taiwan; 4 Oral & Maxillofacial Imaging Center, College of Dental Medicine, Kaohsiung Medical University, Kaohsiung, Taiwan; 5 Division of Oral & Maxillofacial Surgery, Kaohsiung Medical University Hospital, Kaohsiung, Taiwan

**Keywords:** Oral lesions, Oral health

## Abstract

**Introduction:**

A study of the whole spectrum of biopsied head and neck (HN) diseases in Taiwan has not yet been performed. Therefore, the current study aimed to provide updated information about HN lesions in a cohort of referral Taiwanese patients for histopathological examination.

**Methods:**

HN lesions (2000–2011) in patients with records of age, sex, and histological diagnoses were retrieved from the Oral Pathology Department of the institution. These lesions were classified into four main categories: tumor/tumor-like reactive lesions, cystic/pseudocystic lesions, inflammatory/infective lesions, and others/miscellaneous lesions.

**Results:**

A total of 37,210 HN lesions were included in the current study. Most of these lesions were distributed in the group of tumor/tumor-like reactive lesions, followed by the groups of inflammatory/infective lesions, cystic/pseudocystic lesions, and others/miscellaneous lesions. Squamous cell carcinoma was the most common HN lesion, and was also the most frequent malignant lesion among the referral patients.

**Conclusion:**

It was worthy of note that squamous cell carcinoma and oral potentially malignant disorders comprised high percentages of all HN lesions for the present cohort of referral patients.

## Introduction

Reviewing the English literature, to our knowledge, most of the previous studies of HN lesions analyzed specific diseases, such as odontogenic cysts or tumors [1,2], in certain populations, such as pediatric or geriatric populations [3,4]. There are only a few retrospective reports focusing on the prevalence the whole spectrum of biopsied oral and maxillofacial (OMF) lesions in various countries [5-11]. Information of the types and frequency of HN lesions in the local population may always be helpful in management the patients. A study of a variety of biopsied HN diseases in Taiwanese patients has not yet been performed. Hence, the present study aimed to provide updated information about HN lesions in a cohort of referral Taiwanese patients for histopathological examination.

## Materials and methods

The Oral Pathology Department of the institution is the department providing a histopathological service encompassed by HN surgery specialty in southern Taiwan, receiving specimens mainly from the surgeons of the OMF Surgery Department, ENT Department, and Plastic Surgery Department of the hospital as well as other nearby regional hospitals and local dental clinics. Three experienced board-certified HN pathologists make the histological diagnosis for each biopsy independently, based mostly on paraffin embedded sections of hematoxylin-eosin staining and sometimes conjunction with immunohistochemical and/or histochemical staining. The histological diagnoses are established by peer slide review; however, if disagreement exists amongst the pathologists, a consensus is reached upon mutual discussion.

This study complied with the Helsinki Declaration with the data collected after the approval of the Institutional Review Board of the hospital (KMUH-IRB-2014-73). A total of 39,503 diagnosed cases in the HN region between 2000 and 2011 were retrieved from the database of the Oral Pathology Department. With the exclusion of normal tissues and lesions without specific findings, a pool of 37,210 cases was included for analyses. Age, sex, and histological diagnoses were recorded for these HN lesions, which were classified into four main categories: tumor/tumor-like reactive lesions, cystic/pseudocystic lesions, inflammatory/infective lesions, and others/miscellaneous lesions.

Statistical analyses (chi-square test/binominal proportion test) for prevalence rates of the lesions, age and sex distributions of the patients were performed using SAS Statistical Package (Version 9.1.3, SAS Institute Inc., Cary, NC, USA). Since the number of the items of some tables was as large as approximately 50, we employed the Bonferroni method (threshold of p = 0.001; p_0_/N, p_0_ = 0.05, N = 50 items) for multiple testing-adjusted corrections. Hence, the results were considered significant when the p value was < 0.001 (i.e. 0.05/50). If p < 0.0001, the research findings were very highly significant.

## Results

The frequencies of the 12 most common HN lesions, with a total number of 28,783, comprised 77.3% of all the lesions in the current study, are shown in Table 1. The most common disease in the present cohort was squamous cell carcinoma (SCC, 13.3%), followed by hyperkeratosis (HK, 12.8%), epithelial dysplasia (ED, 7.8%), candidiasis (6.8%), oral submucous fibrosis (OSF, 6.7%) and epithelial hyperplasia (EH, 6.4%); the aforementioned first six most common lesions constituted more than 50% of all the HN lesions. With the exception of HK, the percentage of SCC was significant higher than ED, candidiasis, OSF, and EH (p < 0.0001).

**Table 1:** Number and percentages of the 12 most common head & neck lesions

**12 most common lesions**	**Number**	**% of all lesions**
Squamous cell carcinoma	4960	13.3
Hyperkeratosis*	4779	12.8
Epithelial dysplasia*	2899	7.8
Candidiasis	2535	6.8
Oral submucous fibrosis*	2500	6.7
Epithelial hyperplasia*	2371	6.4
Verrucous hyperplasia*	1850	5.0
Inflammation	1821	4.9
Radicular cyst	1720	4.6
Apical granuloma	1395	3.8
Non-specific ulcer	986	2.6
Mucocele	967	2.6
Total number	28783	77.3

*Oral potentially malignant disorders.

Various groups of HN lesions are classified in Table 2. The highest number of lesions were distributed in the group of oral potentially malignant disorders (38.7%), followed by the groups of inflammatory/infective lesions (31.6%) and non-odontogenic malignant lesions (16.2%). In contrast, the lowest number of lesions was found in the non-odontogenic cystic/pseudocystic group, in which only 91 cases were included. The percentage of oral potentially malignant disorders was significant higher than inflammatory/infective lesions and non-odontogenic malignant lesions (p < 0.0001). Also, the percentage of inflammatory/infective lesions was significant higher than and non-odontogenic malignant lesions (p < 0.0001).

**Table 2:** Number and percentages of the four categories of head & neck lesions

**Categories**		**Number**	**% of total**
Tumor/tumor-like reactive lesions	Odontogenic (benign)	456	1.23
	Non-odontogenic (benign)		
	Bone	160	0.43
	Salivary gland	96	0.26
	Epithelial	118	0.32
	Soft tissue	1251	3.36
	Non-odontogenic (oral potentially malignant disorder)		
	Epithelial	14399	38.70
	Non-odontogenic (malignant)		
	Mesenchymal	15	0.04
	Hematologic	45	0.12
	Salivary gland	82	0.22
	Epithelial	5886	15.82
Cystic/pseudocystic lesions	Odontogenic	2274	6.11
	Non-odontogenic	91	0.25
Inflammatory/infective lesions		11775	31.65
Others/miscellanous lesions		562	1.51

The sex and age distribution of the HN lesion patients is shown in Table 3. Most lesions were distributed in the range of 50–59 years, followed by 40–49 years, and 30–39 years, all of which comprised more than 70.0% of all HN lesions. The percentage of the patients in the range of 50–59 years was significantly higher than the ranges of 40–49 years and 30–39 year respectively (p < 0.0001) whilst the percentage of the range of 40–49 year was significant higher than the range of 30–39 year (p < 0.0001). Moreover, in the above three age groups, the number of male patients was higher significantly than the number of female patients, particularly in the highest male to female ratio in the range of 40–49 years (p < 0.0001), whereas the lowest male to female ratio was found in the range of 90–99 years (p = 0.0023). The lowest number of lesions was found in the range of 90–99 years.

**Table 3:** Age and sex distribution of the patients presenting head & neck lesions

**Age (years)**	**Number**	**Male:Female**
0–9	362	1.1:1
10–19	970	1.2:1
20–29	2505	1.6:1
30–39	5733	4.2:1
40–49	9871	5.5:1
50–59	11053	4.4:1
60–69	3400	3.1:1
70–79	2676	1.6:1
80–89	608	0.8:1
90–99	32	0.3:1
**Total**	**37210**	**3.4:1**

The data of the odontogenic cyst group are presented in Table 4, with a male to female ratio of about 1.3:1. (p < 0.0001). Radicular cyst was the most common lesion, comprising about 75.0% of the lesions within this group, and was mostly distributed in the third to the sixth decades of life significantly higher than other groups (p < 0.0001). The second and the third most common lesions in this group were dentigerous cyst and keratocystic odontogenic tumor (formerly called odontogenic keratocyst), respectively; both lesions collectively comprised about 20.0% of the lesions. A significant difference of distribution percentage was found among radicular cyst, dentigerous cyst and keratocystic odontogenic tumor (p < 0.0001).

**Table 4:** Number, sex, and age distribution of the patients presenting odontogenic cysts

**Odontogenic cysts/pesudocysts**	**Male**	**Female**	**Age distribution (years)**									
			**0-9**	**10-19**	**20-29**	**30-39**	**40-49**	**50-59**	**60-69**	**70-79**	**80-89**	**90-99**
Radicular cyst	910	810	15	98	313	343	368	374	79	105	25	0
Dentigerous cyst	200	98	28	65	54	39	53	38	8	11	2	0
Keratocystic odontogenic tumor	106	64	4	40	46	34	25	14	2	3	2	0
Residual cyst	28	20	2	3	1	10	3	17	2	10	0	0
Calcifying odontogenic cyst	13	5	1	7	3	2	3	0	1	1	0	0
Paradental cyst	4	3	0	0	4	2	1	0	0	0	0	0
Glandular odontogenic cyst	4	0	0	0	0	1	1	0	2	0	0	0
Eruption cyst	4	0	1	3	0	0	0	0	0	0	0	0
Lateral periodontal cyst	2	2	0	0	1	0	0	1	2	0	0	0
Gingival cyst	1	0	1	0	0	0	0	0	0	0	0	0
**Total**	**1272**	**1002**	**52**	**216**	**422**	**431**	**454**	**444**	**96**	**130**	**29**	**0**

Only 91 cases were contained in the group of non-odontogenic cystic/pseudocystic lesions (Table 5), with a significant difference of male to female ratio of 2.8:1 (p < 0.0001). The most common lesion in this group was epidermoid cyst, followed by lymphoepithelial cyst and nasopalatine duct cyst. The percentage of epidermoid cyst was significant higher than lymphoepithelial cyst and nasopalatine duct cyst (p < 0.0001) whilst the percentage of lymphoepithelial cyst and nasopalatine duct cyst was similar to each other (p < 0.4328). Most of the epidermoid cysts were distributed in the range of 40–49 years, and the number of male patients was much higher than that of female patients (p < 0.0001); the second (lymphoepithelial cyst) and the third (nasopalatine duct cyst) most common lesions were mostly found in the first four decades of life.

**Table 5:** Number, sex, and age distribution of the patients presenting non-odontogenic cyst/pesudocysts

**Non-odontogenic cyst/pesudocysts**	**Male**	**Female**	**Age distribution (years)**									
			**0-9**	**10-19**	**20-29**	**30-39**	**40-49**	**50-59**	**60-69**	**70-79**	**80-89**	**90-99**
Epidermoid cyst	34	7	0	2	4	6	16	6	3	3	1	0
Lymphoepithelial cyst	9	6	0	1	2	4	3	3	1	1	0	0
Nasopalatine duct cyst	7	4	0	0	2	2	1	4	2	0	0	0
Simple bone cyst	2	3	0	3	0	1	1	0	0	0	0	0
Aneurysmal bone cyst	4	0	0	0	3	0	0	1	0	0	0	0
Sebaceous cyst	2	1	0	0	2	0	1	0	0	0	0	0
Nasolabial cyst	2	1	0	0	0	0	1	2	0	0	0	0
Thyroglossal duct cyst	3	0	0	0	0	0	2	0	1	0	0	0
Incisive canal cyst	2	2	0	0	3	1	0	0	0	0	0	0
Branchial cleft cyst	1	0	0	0	0	0	0	0	0	1	0	0
Globulomaxillary cyst	1	0	0	0	0	1	0	0	0	0	0	0
**Total**	**67**	**24**	**0**	**6**	**16**	**15**	**25**	**16**	**7**	**5**	**1**	**0**

The data of benign odontogenic tumors are listed in Table 6, with a male to female ratio of almost 1:1 (p = 0.9254). The most common lesion within this group was odontoma, comprising about 40.0% of the lesions, which was predominantly found in the range of 10–19 years compared with the other groups (p < 0.0001). The second most common lesion was ameloblastoma, which was mostly distributed in the second and the third decade of life. There were no significant differences between odontoma and ameloblastoma (p *=* 0.0557).

**Table 6:** Number, sex, and age distribution of the patients presenting benign odontogenic tumors

**Odontogenic (benign) tumors**	**Male**	**Female**	**Age distribution (years)**									
			**0-9**	**10-19**	**20-29**	**30-39**	**40-49**	**50-59**	**60-69**	**70-79**	**80-89**	**90-99**
Odontoma	92	103	37	94	29	14	7	12	2	0	0	0
Ameloblastoma	92	67	5	14	42	35	24	27	11	1	0	0
Odontogenic fibroma	17	29	4	8	12	9	5	4	2	2	0	0
Odontogenic myxoma	11	9	0	8	5	1	4	0	0	2	0	0
Cementoblastoma	3	6	0	1	0	2	3	1	1	0	1	0
Adenomatoid odontogenic tumor	2	6	0	4	0	2	2	0	0	0	0	0
Ameloblastic odontoma	6	2	2	6	0	0	0	0	0	0	0	0
Calcifying epithelial odontogenic tumor	0	5	0	0	1	0	4	0	0	0	0	0
Ameloblastic fibroma	3	1	3	1	0	0	0	0	0	0	0	0
Squamous odontogenic tumor	1	1	1	0	0	0	0	0	1	0	0	0
**Total**	**227**	**229**	**52**	**136**	**89**	**63**	**49**	**44**	**17**	**5**	**1**	**0**

Most of the HN lesions in the current study were distributed in the group of benign non-odontogenic tumor/tumor-like reactive lesions (Table 7). The most common lesion of this group was noted in the epithelial subgroup, followed by the soft tissue subgroup, bone subgroup, and salivary gland subgroup. The percentage of the epithelial subgroup (n = 14,517) was significantly higher than the soft tissue subgroup (n = 1,251), bone subgroup (n = 160), and salivary gland subgroup (n = 96) (p < 0.0001). Also, the percentage of the soft tissue subgroup (n = 1251) was significantly higher than the bone subgroup (n = 160), and salivary gland subgroup (n = 96) (p < 0.0001). The percentage of the bone subgroup (n = 160) was significantly higher than the salivary gland subgroup (n = 96) (p < 0.0001).

**Table 7:** Number, sex and age distribution of patients presenting benign non-odontogenic tumor/tumor-like reactive lesions

**Non-odontogenic (benign) tumor/tumor-like reactive lesions**	**Male**	**Female**	**Age distribution (years)**									
			**0-9**	**10-19**	**20-29**	**30-39**	**40-49**	**50-59**	**60-69**	**70-79**	**80-89**	**90-99**
**Bone**												
Cemento-ossifying fibroma	18	40	0	2	9	22	15	8	0	2	0	0
Cemental-osseous dysplasia	2	33	0	4	4	3	9	10	4	1	0	0
Fibrous dysplasia	11	18	0	2	12	8	4	3	0	0	0	0
Osteoma	7	9	0	3	4	1	3	2	2	1	0	0
Central neurofibroma	1	3	0	0	0	2	0	2	0	0	0	0
Osseous choristoma	0	3	0	0	0	1	2	0	0	0	0	0
Central giant cell granuloma	2	1	1	0	1	0	0	1	0	0	0	0
Osteoblastoma	0	3	0	2	0	0	1	0	0	0	0	0
Central schwannoma	1	2	0	0	0	0	2	1	0	0	0	0
Desmoplastic fibroma	0	1	0	0	0	0	1	0	0	0	0	0
Osteolipoma	0	1	0	0	0	0	0	0	0	1	0	0
Chondroid choristoma	0	1	0	0	0	0	0	0	0	0	1	0
Ossifying fibromyxoid tumor	0	1	0	0	0	0	1	0	0	0	0	0
Juvenile aggressive ossifying fibroma	0	1	0	0	1	0	0	0	0	0	0	0
Synovial chondromatosis	0	1	0	0	0	0	0	0	1	0	0	0
**Salivary gland**												
Pleomorphic adenoma	32	33	0	0	8	18	16	13	8	2	0	0
Warthin's tumor	10	2	0	0	0	0	2	7	2	1	0	0
Papillary cystic adenoma	4	4	0	0	3	0	0	2	0	3	0	0
Necrotizing sialometaplasia	3	1	0	0	0	1	1	1	1	0	0	0
Adenomatoid hyperplasia	2	0	0	0	1	0	0	1	0	0	0	0
Oncocytosis	1	1	0	0	0	0	1	1	0	0	0	0
Tubular adenoma	2	0	0	0	0	0	0	2	0	0	0	0
Benign lymphoepithelial lesion	1	0	0	0	0	0	0	0	0	1	0	0
**Epithelial**												
Hyperkeratosis*	4294	485	4	29	252	938	1425	1444	438	216	31	2
Epithelial dysplasia*	2648	251	0	0	60	374	765	1053	364	237	44	2
Oral submucous fibrosis*	2330	170	0	11	171	534	741	701	247	93	2	0
Epithelial hyperplasia*	2088	283	3	14	95	376	646	801	243	169	23	1
Verrucous hyperplasia*	1726	124	1	2	33	308	572	599	157	169	8	1
Papilloma	74	41	0	4	12	15	31	33	7	9	4	0
Seborrheic keratosis	2	1	0	0	0	2	0	1	0	0	0	0
**Soft tissue**												
Fibroma	311	314	20	20	64	112	158	170	35	33	12	1
Hemangioma	99	85	5	13	11	29	35	53	20	13	4	1
Fibrous hyperplasia	98	83	0	3	5	8	16	19	50	54	26	0
Lipoma	22	15	0	1	2	2	9	7	10	6	0	0
Verruca vulgaris	14	21	0	1	5	8	12	9	0	0	0	0
Peripheral odontogenic fibroma	7	22	0	1	5	7	10	6	0	0	0	0
Nevus	11	14	1	1	8	7	4	3	1	0	0	0
Lymphangioma	13	12	2	5	3	2	2	4	4	3	0	0
Verruciform xanthoma	17	3	0	0	1	3	4	8	2	2	0	0
Myxofibroma	3	11	0	0	3	3	3	2	2	1	0	0
Schwannoma	5	6	1	4	2	3	0	1	0	0	0	0
Neurofibroma	7	5	0	0	2	0	5	4	1	0	0	0
Neuroma	3	5	0	1	0	1	1	3	2	0	0	0
Xanthogranuloma	2	5	0	0	1	1	0	5	0	0	0	0
Hemangioendothelioma	5	0	0	0	1	1	1	2	0	0	0	0
Granular cell tumor	2	3	0	0	3	1	1	0	0	0	0	0
Sclerosing hemangioma	2	2	0	0	0	0	3	1	0	0	0	0
Peripheral giant cell granuloma	3	0	0	0	0	0	1	1	0	0	1	0
Myofibroma	0	2	0	0	0	1	0	1	0	0	0	0
Keratoacanthoma	0	2	0	0	0	0	0	0	2	0	0	0
Peripheral odontogenic myxoma	1	1	0	0	0	0	0	0	1	1	0	0
Peripheral ossifying fibroma	0	2	0	0	0	0	1	1	0	0	0	0
Lipofibroma	1	1	0	0	0	0	1	1	0	0	0	0
Oral focal mucinosis	1	1	0	0	0	0	1	0	1	0	0	0
Neurofibromatosis	1	0	0	0	0	0	0	1	0	0	0	0
Angiofibroma	1	0	0	0	0	0	0	1	0	0	0	0
Fibrolipoma	1	0	0	0	0	0	1	0	0	0	0	0
Leiomyoma	1	0	0	0	0	0	0	1	0	0	0	0
Fibrous histiocytoma	1	0	0	0	0	0	1	0	0	0	0	0
Angiomyxoma	0	1	0	1	0	0	0	0	0	0	0	0
Lipoblastoma	1	0	0	1	0	0	0	0	0	0	0	0
Solitary fibrous tumor	0	1	0	0	0	1	0	0	0	0	0	0
Hemangiolymphangioma	0	1	0	0	1	0	0	0	0	0	0	0
**Total**	**13892**	**2132**	**38**	**125**	**783**	**2793**	**4508**	**4990**	**1605**	**1018**	**156**	**8**

*Oral potentially malignant disorder.

Five of the seven types of lesions in the epithelial subgroup were potentially malignant disorders, and all these five lesions belonged to the top 12 diseases. The most common lesion of the potentially malignant disorders was HK, followed by ED and OSF. The number of male patients was almost ten times that of the female patients in all the lesions of potentially malignant disorders (p < 0.0001).

Fibroma, comprising about 50% of the lesions, was the most common lesion in the soft tissue subgroup, significantly higher than the other groups (p < 0.0001), with the number of male patients almost equal to that of female patients (p *=* 0.9045). Hemangioma was the second most common lesion, which was mostly found in the sixth decade of life. In the hemangioma, the percentage of sixth decade of life was observed no significantly difference than the age 40–49 (p = 0.0550). The 50–59 age group compared with other groups, the p value was < 0.0001, except for 30–39 age group (p = 0.0080), and 60–69 age group (p = 0.0001).

Cemento-ossifying fibroma, cemento-osseous dysplasia, and fibrous dysplasia, comprising about 76.0% of the lesions, were the three most common lesions within the bone subgroup, in which the number of males was lower than that of females. The percentage of cemento-ossifying fibroma was observed higher than cemento-osseous dysplasia (p = 0.0171). Compared with fibrous dysplasia, cemento-ossifying fibroma had higher percentage than fibrous dysplasia (p = 0.0019). There were no difference of percentage between cemento-osseous dysplasia and fibrous dysplasia (p = 0.4533). In cemento-ossifying fibroma, the percentage of males was significantly lower than females (p = 0.0039). In cemento-osseous dysplasia, the percentage of males was significantly lower than females (p < 0.0001). In fibrous dysplasia, the percentage of males and females was similar (p = 0.1936).

Pleomorphic adenoma was the most common lesion in the salivary gland subgroup compared with the other type (p < 0.0001), with an almost equal sex distribution (p = 0.9013); this comprised about 67.0% of the lesions and was mostly noted in the fourth to the sixth decades of life. Warthin’s tumor was the second most common lesion, in which the number of males was much higher than that of females (p = 0.0209).

The data of malignant non-odontogenic tumor/tumor-like reactive lesions are shown in Table 8, with the number of male patients being much higher than that of female patients (12:1) (p < 0.0001); this was mostly located in the fifth and sixth decades of life compared with the other the age groups (p < 0.0001). The highest number of lesions was noted in the epithelial subgroup, in which SCC predominated (82.0%), followed by verrucous carcinoma (6.5%). SCC had significant higher percentage than the other types (p < 0.0001). The second highest number of lesions was found in the salivary gland subgroup, with mucoepidermoid carcinoma and adenoid cystic carcinoma, comprising about 77.0% of all lesions, being the two most common lesions. Mucoepidermoid carcinoma had significant higher percentage than the other types (p < 0.0001). Adenoid cystic carcinoma had higher percentage than the other types (p = 0.0009). On the other hand, the lowest number of lesions was found in the mesenchymal subgroup, the most frequent lesion being osteosarcoma, which comprised about 60.0% of the lesions. Osteosarcoma had higher percentage than malignant fibrous histiocytoma (p = 0.0348). The second lowest number of lesions was noted in the hematologic subgroup, in which non-Hodgkin’s lymphoma and Langerhans cell histocytosis, comprising about 81.5% of the lesions, were the two most frequent lesions.

**Table 8:** Number, sex and age distribution of patients presenting malignant non-odontogenic tumors

**Non-odontogenic (malignant) tumors**	**Male**	**Female**	**Age distribution (years)**									
			**0-9**	**10-19**	**20-29**	**30-39**	**40-49**	**50-59**	**60-69**	**70-79**	**80-89**	**90-99**
**Mesenchymal**												
Osteosarcoma	3	6	0	3	0	0	0	3	1	1	1	0
Malignant fibrous histiocytoma	2	0	0	0	0	0	0	2	0	0	0	0
Malignant solitary fibrous tumor	1	0	0	0	0	1	0	0	0	0	0	0
Neuroblastoma	0	1	1	0	0	0	0	0	0	0	0	0
Ewing sarcoma	1	0	0	1	0	0	0	0	0	0	0	0
Chondrosarcoma	0	1	0	0	0	0	0	1	0	0	0	0
**Hematologic**												
Non-Hodgkin's lymphoma	13	6	0	1	3	0	5	5	0	4	1	0
Langerhans cell histocytosis	12	0	0	0	2	6	2	2	0	0	0	0
Plasmacytoma	3	4	0	0	0	0	5	2	0	0	0	0
Leukemia	0	3	2	0	0	0	1	0	0	0	0	0
Hodgkin's lymphoma	1	1	0	0	0	0	0	0	0	2	0	0
Malignant hemangioendothelioma	0	1	0	0	1	0	0	0	0	0	0	0
Multiple myeloma	0	1	0	0	0	0	0	0	0	1	0	0
**Salivary gland**												
Mucoepidermoid carcinoma	19	16	0	0	7	9	7	6	0	3	3	0
Adenoid cystic carcinoma	18	10	0	0	1	4	3	5	0	12	3	0
Papillary cystic adenocarcinoma	6	2	0	0	0	0	0	0	0	6	2	0
Adenocarcinoma, NOS	4	3	0	0	2	0	2	3	0	0	0	0
Salivary duct carcinoma	2	1	0	0	1	2	0	0	0	0	0	0
Acinic cell carcinoma	1	0	0	0	0	0	0	0	0	1	0	0
**Epithelial**												
Squamous cell carcinoma	4628	332	0	0	49	603	1709	1735	439	350	71	4
Verrucous carcinoma	359	34	0	1	0	42	113	136	40	52	9	0
Metastatic carcinoma	319	29	0	0	0	46	137	128	22	14	1	0
Carcinoma in situ	142	13	0	0	1	23	40	53	18	16	4	0
Undifferentiated carcinoma	11	4	0	0	0	2	2	3	2	3	3	0
Spindle cell carcinoma	10	1	0	0	0	4	1	4	0	2	0	0
Carcinosarcoma	3	1	0	0	0	1	2	1	0	0	0	0
**Total**	**5558**	**470**	**3**	**6**	**67**	**743**	**2029**	**2089**	**522**	**467**	**98**	**4**

The second greatest number of HN lesions was noted in the inflammatory/infective group (Table 9), which comprised about 31.8% of all the HN lesions, with a male to female ratio of about 1.7:1 (p < 0.0001). Candidiasis was the most common lesion in this group, followed by inflammation, with both lesions being located in the range of 50–59 years. In candidiasis, the 50–59 years had significant higher percentage than the other age groups (p < 0.0001). In inflammation, the 50–59 years had significant higher percentage than the other age groups (p < 0.0001), except for 40–49 age group (p = 0.0112). Apical granuloma, distributed evenly in the fourth to the sixth decades of age, was the third most common lesion in this group.

**Table 9:** Number, sex and age distribution of patients presenting inflammatory/infective lesions

**Inflammatory/infective lesions**	**Male**	**Female**	**Age distribution (years)**									
			**0-9**	**10-19**	**20-29**	**30-39**	**40-49**	**50-59**	**60-69**	**70-79**	**80-89**	**90-99**
Candidiasis	1908	627	13	8	52	290	614	872	328	275	79	4
Inflammation	1149	672	21	55	134	191	434	512	191	196	83	4
Apical granuloma	551	844	0	49	242	312	340	320	55	66	10	1
Non-specific ulcer	728	258	6	21	46	103	210	290	162	105	43	0
Mucocele	539	428	103	179	320	161	91	66	25	19	3	0
Lymphadenitis	539	43	1	1	14	86	228	179	62	9	2	0
Pyogenic granuloma	323	189	8	13	44	93	128	134	43	40	8	1
Granulation tissue	307	196	12	37	38	57	108	136	46	55	13	1
Lichen planus*	201	268	0	5	28	59	121	177	45	30	4	0
Osteomyelitis	141	145	0	3	10	19	45	76	39	64	25	5
Sequestrum	149	113	1	2	6	20	39	72	44	50	25	3
Sialadenitis	87	103	2	2	11	29	45	66	9	21	5	0
Scar tissue	145	32	2	9	7	26	49	49	15	16	3	1
Inflammatory fibrous hyperplasia	94	69	0	0	14	27	40	82	0	0	0	0
Apical scar	63	69	0	2	26	36	36	32	0	0	0	0
Acanthosis	89	12	0	2	5	30	27	37	0	0	0	0
Necrotic tissue	72	18	3	2	4	4	24	35	10	6	2	0
Mucositis	56	27	1	1	7	16	17	26	11	4	0	0
Osteoradionecrosis	53	4	0	0	0	3	11	30	4	7	2	0
Fibrosis	28	19	1	3	5	7	10	19	0	2	0	0
Foreign body granuloma	33	13	0	0	0	6	19	18	0	1	2	0
Ranula	20	18	0	2	16	6	3	11	0	0	0	0
Sinusitis	22	11	0	5	5	4	8	8	2	1	0	0
Sialoithiasis	20	9	0	0	8	2	4	10	1	4	0	0
Gingival hyperplasia	14	10	2	3	8	6	1	0	1	3	0	0
Osteosclerosis	9	14	0	1	7	3	5	7	0	0	0	0
Tuberculosis	16	5	3	0	2	3	2	4	2	3	2	0
Epulis granulomatosum	6	14	0	0	2	5	1	6	1	2	3	0
Pemphigus vulgaris*	14	6	0	1	0	1	9	5	4	0	0	0
Actinomycosis	9	8	0	2	0	2	2	5	1	5	0	0
Sjogren syndrome*	1	14	0	1	1	2	4	3	1	3	0	0
Periodontitis	8	6	0	1	1	1	5	3	1	2	0	0
Condensing osteitis	7	6	0	0	0	0	2	5	4	2	0	0
Gingivitis	7	5	0	0	0	3	2	5	2	0	0	0
Fistula	10	2	0	1	0	1	6	2	1	1	0	0
Pseudoepitheliomatous hyperplasia	6	2	0	0	0	0	2	6	0	0	0	0
Epulis fissuratum	5	3	0	0	0	0	0	0	6	2	0	0
Periapical abscess	6	1	0	0	0	0	0	6		1	0	0
Glossitis	0	6	0	0	0	1	0	3	0	2	0	0
Thrombus	3	3	0	1	0	0	2	1	2	0	0	0
Polyp	4	2	0	0	2	0	1	2	1	0	0	0
Pulp stone	2	2	0	0	2	0	0	2	0	0	0	0
Pulpitis	2	2	0	0	0	0	0	2	2	0	0	0
Plasma cell granuloma	2	2	0	0	1	1	2	0	0	0	0	0
Amyloidosis	3	0	0	0	0	0	0	1	1	0	1	0
Pemphigoid	0	3	0	0	0	1	1	1	0	0	0	0
Paget's disease	2	0	0	0	0	0	0	2	0	0	0	0
Histioplasmosis	1	1	0	0	0	0	0	1	0	1	0	0
Herpes simplex infection	2	0	0	0	0	0	0	2	0	0	0	0
Pericornitis	2	0	0	0	0	0	0	2	0	0	0	0
Myositis	1	0	1	0	0	0	0	0	0	0	0	0
Phlebolith with thrombosis	0	1	0	0	0	0	0	0	1	0	0	0
Psoriasiform mucositis	1	0	0	0	0	0	1	0	0	0	0	0
Acanthosis nigricans	1	0	0	0	0	0	1	0	0	0	0	0
Tonsilitis	0	1	0	0	0	1	0	0	0	0	0	0
Chondroid hyperplasia	1	0	0	0	0	1	0	0	0	0	0	0
Nodular fascitis	1	0	0	0	0	0	0	1	0	0	0	0
Socket sclerosis	0	1	0	0	0	0	0	0	0	1	0	0
Caries	1	0	0	0	0	0	0	0	0	1	0	0
Varicosities	0	1	0	0	0	0	0	1	0	0	0	0
Lupus erythematosus	1	0	0	0	0	1	0	0	0	0	0	0
Sialodochitis	1	0	0	1	0	0	0	0	0	0	0	0
Phycomycosis	0	1	0	1	0	0	0	0	0	0	0	0
**Total**	**7466**	**4309**	**180**	**414**	**1068**	**1620**	**2700**	**3335**	**1123**	**1000**	**315**	**20**

*Autoimmune disease.

The data of the others/miscellaneous lesions are shown in Table 10, with the number of female patients being slightly higher than that of the male patients (p = 0.8660). Exostosis, mostly distributed in the range of 50–59 years, was the most common lesion within this group and comprised about 44.0% of the lesions. The exostosis had the highest percentage than other types significantly (p < 0.0001). Dense fibrotic dental follicle was the second most common lesion, which occurred mostly in the first three decades of life. The dense fibrotic dental follicle had the highest percentage than the other types significantly (p < 0.0001).

**Table 10:** Number, sex and age distribution of patients presenting others/miscellaneous lesions

**Others/miscellanous lesions**	**Male**	**Female**	**Age distribution (years)**									
			**0-9**	**10-19**	**20-29**	**30-39**	**40-49**	**50-59**	**60-69**	**70-79**	**80-89**	**90-99**
Exostosis	119	142	1	4	9	41	56	99	14	34	3	0
Dense fibrotic dental follicle	57	59	26	50	30	5	3	2	0	0	0	0
Foreign body	34	15	0	2	3	3	18	15	5	3	0	0
Hematoma	20	15	0	1	2	3	7	10	4	6	2	0
Melanotic macule	8	17	0	0	5	6	3	9	0	1	1	0
Fordyce's granule	12	6	0	1	1	2	10	3	1	0	0	0
Hypercementosis	6	5	0	0	1	1	3	2	3	1	0	0
Supernumery tooth	5	5	3	3	2	1	0	1	0	0	0	0
Blood clot	6	3	0	1	1	0	1	1	1	2	2	0
Melanoplakia	3	4	0	1	2	3	0	1	0	0	0	0
Internal root resorption	1	3	0	2	1	0	0	0	0	1	0	0
Mesioden	1	3	2	2	0	0	0	0	0	0	0	0
Calcification	2	1	0	0	0	0	1	1	1	0	0	0
Osteoporosis	2	0	0	0	0	0	0	0	0	2	0	0
Dentinogenesis imperfecta	0	2	2	0	0	0	0	0	0	0	0	0
Melanosis	1	1	2	0	0	0	0	0	0	0	0	0
Leukoedema	1	0	0	0	0	1	0	0	0	0	0	0
Amalgum tatoo	0	1	0	0	0	0	0	0	1	0	0	0
Epithelial atrophy	1	0	0	0	0	0	0	0	0	1	0	0
Natal tooth	0	1	1	0	0	0	0	0	0	0	0	0
**Total**	**279**	**283**	**37**	**67**	**57**	**66**	**102**	**144**	**30**	**51**	**8**	**0**

## Discussion

The Oral Pathology Department not only provides services for nearly all the biopsied HN lesions (Medical Pathology contributes only a minor number of cases), but is also the most heavily used referral center for patients with these lesions in southern Taiwan. Therefore, the various types of lesions in the current study are representative of the occurrence of such lesions among the cohort of referral patients for a biopsy procedure in this geographical region.

In the current study, a total of 37,210 cases of HN lesions from 2000–2011 was documented in a cohort of Taiwanese patients referred for histopathological examination. SCC was the most common lesion in this cohort (13.3%), which was in contrast to the findings of Franklin & Jones [8] and Ali & Sundaram [9], in which fibrous hyperplasia (14.7% and 20.7% respectively) was the most common lesion, as well as different to the findings of Bhasker [10] and Tay [11], in which dental granuloma (11.1%) and fibrous epulis (10.3%) were respectively the most frequent lesions. This may be due to the fact that betel quid chewing, which is a high risk factor for oral epithelial malignancies, has a high incidence in Taiwan [12]. This finding was consistent to the high incidence (16.2–39.0%) of oral cancers in India [13], in which betel quid chewing has been responsible for half of oral cancer cases [14]. On the other hand, the prevalence of OSF, being one of the oral potential malignant disorders closely associated with betel quid chewing, of the present study (6.7%) was compatible to that of the study from India (7.1%) [15]. So, the high frequency of betel quid chewing in Taiwan may also contribute to the finding that five of the twelve most common lesions were potentially malignant disorders in the present study. However, it should be cautioned that the present cohort consisted only of patients and such patients were much more likely to be referred for histopathologcal assessments; hence, a higher prevalence of SCC and oral potentially malignant disorders would be expected to be observed.

Most lesions in the current study were distributed in the benign non-odontogenic tumor group (43.1%), followed by the inflammatory/infective group (31.6%), which was compatible with the findings of Jones & Franklin [6]. On the other hand, the lowest numbers of lesions was in the non-odontogenic cyst/pseudocyst group, which was consistent with the other studies [7,10,11]. Moreover, oral epithelial malignant lesions comprised 16.2% of all HN lesions in the present cohort, which was appeared to be much higher than the data of Bhaska (6.9%) [10].

The overall male to female ratio (3.4:1) in the current study was higher than the studies of Ali (1.1:1) [7], Ali & Sundaram (1.1:1) [9], Akinmoladun et al. (1.2:1) [5], and Jones & Franklin (0.9:1) [6]. Most lesions were located in the range of 50–59 years, followed by 40–49 years, which may be attributed to the aging population becoming dominant in Taiwan [16]. Additionally, this finding was in contrast to the study of Akinmoladun et al. in Nigeria (range: 20–29 years) [5].

The lesions in the odontogenic cystic group were mostly distributed in the third to the sixth decade of life, and the number of males was slightly higher than the number of females; this result was compatible with the study of Avelar et al. [1]. Radicular cyst was the most common (76.0%) of the lesions within the odontogenic cyst group in this study; this finding was similar to other studies [17-21]. Furthermore, most radicular cysts were distributed in the maxilla (64.7%) in this cohort, which was similar to the results of Souza et al. (63.0%) [21] and Prockt et al. (66.0%) [20]. Dentigerous cyst was the second most common lesion (13.1%), in which the ratio of males to females was about 2:1; this was similar to the study of Selvamani et al. [18]. More cases of dentigerous cyst were noted in Bhasker’s study (33.8%) [10] in comparison with the present study cohort. Additionally, although the majority of dentigerous cysts were located in the mandible (62.4%) in this study, this was lower than the findings of Souza et al. (81.0%) [21] and Prockt et al. (69.0%) [20]. Keratocystic odontogenic tumor was the third most common lesion in this group (7.0%), which was compatible with the study of Jones & Franklin [6]; however, this lesion was the most frequent in the study of Koivisto et al. [22]. Moreover, most keratocystic odontogenic tumors were located in the mandible in this study cohort, similar to the results of the other studies [18,19,21,22].

Epidermoid cyst was the most common lesion in the group of non-odontogenic cyst/pseudocyst in the current cohort, which was in contrast to the studies of Ali [7], Bhasker [10] and Tay [11], in which nasopalatine duct cyst was found to be the most common lesion respectively in these three studies. Lymphoepithelial cyst and nasopalatine duct cyst were the second and the third most common lesions in this group, but the numbers of cases of these two kinds of cyst were much lower than that of epidermoid cyst.

Odontoma was the most common lesion in the group of benign odontogenic tumor, constituting about 43.0% of the lesions, which was in contrast to the study of Ali [7]. The ratio of compound to complex odontomas in the current study (1.8:1) was compatible with the results of Bhaskar (2.1:1) [10] and Tay (2.4:1) [11] but in contrast to the data of Jones & Franklin (1:1.6) [6] and Luo & Li (1:1.4) [23]. Furthermore, most of the odontomas were diagnosed in the second decade of life in the study, which was the same as the result of Luo & Li [23]. Although the majority of odontomas in the present study were located in maxilla (56.5%), this was lower than the results of Servato et al. (66.6%) [2] and Luo & Li (66.0%) [23]. Ameloblastoma was the second most common lesion in the study, with a male to female ratio of 1.37:1, which was compatible with the study of Siriwardena et al. (1.03:1) [24], who reported the average age to be 37.5 years, which was also compatible with the study (36.6 years). Additionally, similar to the other studies [2,24-26], most ameloblastomas in the present study were located in the mandible. Odontogenic fibroma was the third most common lesion in this group, which was different to the studies of Bhaskar [10] and Tay [11], who reported that it was the most common lesion. Most of the odontogenic fibromas in our cohort were diagnosed in the third decade of life, which was the same as the studies of Luo & Li [23] and Servato et al. [2].

Pleomorphic adenoma (67.0%) and Warthin’s tumor (12.5%) were the two most common lesions in the salivary gland subgroup, which was compatible with the results of Jaafari-Ashkavandi et al. (80.2% and 10.5%, respectively) [27] and Wang et al. (52.7% and 17.4%, respectively) [28]. Most pleomorphic adenomas in the present study were located in the palate (60.0%), followed by the parotid gland (30.0%), which was different to the studies of Jaafari-Ashkavandi et al. [27] and Wang et al. [28], in which most cases were located in the parotid gland (52.2% and 52.7%, respectively). Additionally, the male to female ratio of the cases of pleomorphic adenoma was almost 1:1 in the study, which was compatible with the report of Jaafari-Ashkavandi et al. (1.26:1) [27]. On the other hand, same as the study of Wang et al. [28], most Warthin’s tumors in the present cohort were located in the parotid gland. The male to female ratio of Warthin’s tumors in the cohort was 5:1, which was higher than the data of Wang et al. [28].

Fibroma was the most common lesion (69.4%) in the soft tissue subgroup, which was compatible with the studies of Ali & Sundaram [9] and Jones & Franklin [6]. Most of the fibromas in the current study were found in the buccal mucosa; however, they most commonly occurred in the gingiva/alveolar ridge in the study of Ali & Sundaram [9]. Hemangioma was the second most common lesion in the study, comprising cavernous (60.0%) and capillary (40%) types, which was compatible with the results of Jones & Franklin (62.0% and 38.0%, respectively) [6]. Furthermore, most of the hemangiomas in the study were noted in the tongue, but no specific location has been documented in other studies [5-11].

In the current study, the number of female patients was about three times higher than that of male patients (1 : 3) in the bone subgroup, which was similar to the result of Ali (1:8) [7]. Cemento-ossifying fibroma was the most common lesion within this subgroup, which was in contrast to the results of Ali [7], who reported central giant cell granuloma to be the most common lesion. Most cemento-ossifying fibromas were located in the mandible, and were diagnosed most frequently in the fourth decade of life in the current study, which was the same as the data of MacDonald-Jankowski [29]. Cemento-osseous dysplasia was the second most common lesion, most documented in the fifth and sixth decades of life, and mostly in females; all these findings were compatible with the study of Alsufyani & Lam [30]. Additionally, most of these lesions were located in the mandible (83.0%), which was similar to the study of Alsufyani & Lam (81.4%) [30].

The lesions within the epithelial subgroup comprised about 39.0% of all HN lesions in the present cohort, which was much higher than the result of Amarasinghe et al. (11.3%) in Sri Lanka [31]. Furthermore, the number of males was much greater than that of females in our study, which was compatible with the reports of Ali & Sundaram [9] and Thomas et al. [32]; however, an almost equal sex distribution was reported in the study of Jones & Franklin [6]. Worthy of note, five of the seven types of lesions in this subgroup were potentially malignant disorders, and were also categorized within the 12 most common lesions; this may be due to the high frequency of oral habits related to risk of oral malignancy in Taiwan. Most of these five potentially malignant disorders were documented in the fifth and sixth decades of life, and most were found in the buccal mucosa, which was compatible with the study of Thomas et al. [32]. HK was the most common lesion in the epithelial subgroup, followed by ED in the current study, which was compatible with the report of Jones & Franklin [6].

Compatible with the study of Ali [7], the number of lesions in the bone subgroup in this study was low, osteosarcoma being the most common lesion, but only nine cases were documented. On the other hand, only 0.7% of lesions were noted in the hematologic subgroup, which was lower than the study of Jones & Franklin [6].

Mucoepidermoid carcinoma and adenoid cystic carcinoma were the two most common malignant lesions in the salivary gland subgroup, which was similar to the results of Bradley & McGurk [33] and Wang et al. [28] but in contrast with the findings of Jaafari-Ashkavandi et al. [27]. The male to female ratio (1.6:1) in this subgroup was compatible with the results of Zohreh et al. (1.2:1) [27] and Wang et al. (1.2:1) [28]. Most mucoepidermoid carcinomas were located in the hard palate and soft palate, which was different to other studies [27,28,33]. Additionally, the mean age at which mucoepidermoid carcinoma was diagnosed in our study (37.6 years) was compatible with the study of Wang et al. (39.5 years) [28]. On the other hand, most of the adenoid cystic carcinomas in our cohort were located in the submandibular gland, which was similar to the studies of Bradley et al. [33] and Wang et al. [28]. Moreover, the mean age (60.5 years) at which adenoid cystic carcinoma was diagnosed in the present study was seemed to be greater than the result of Wang et al. (46 years) [28].

SCC was the most common HN lesion (13.3%) and also the most frequent lesion in the epithelial subgroup of malignant non-odontogenic tumors, which was appeared to be higher than the findings of Jones & Franklin [6] and Tay (3.5%) [11]. Most of the oral epithelial malignant lesions in the current study were located in the buccal mucosa, which was different to the results of Hernandez-Guerrero et al. [34] from Mexico, in which tongue cancer was the most commonly documented malignant lesion. The ratio of males to females in the cohort was 13.9:1, which was seemed to be much higher than the findings of the Internal Agency for Research On Cancer (3.3: 1) [35], Hernandez-Guerrero et al. (1.4 : 1) [34] and Ferlay et al. [36]. Most of the SCCs in the study were distributed in the fifth and sixth decades of life, and the average age was 51.2 years; this result was largely compatible with the seventh and eighth decades of life reported in a North American population [37,38]. The proportion of SCC in patients less than 40 years of age was about 11% in this study, which was compatible with the finding in Indians [39]; moreover, the trend of patients of a younger age with oral cancer has also been documented in the studies from Scotland [40] and the UK [41].

Candidiasis comprised 6.8% of all HN lesions, which was appeared to be higher than the result of Jones & Franklin (1.0%) [6]. The male to female ratio of this lesion (3:1) in the cohort was seemed to be higher than that of Jones & Franklin (1.69:1) [6]. Moreover, most cases of candidiasis were located in the buccal mucosa in the current study, followed by the tongue, which was the same as the study of Ali & Sundaram [9]. Inflammation comprised 4.9% of all HN lesions, which was appeared to be greater than the studies of Tay (3.6%) [11] and Jones & Franklin (1.2%) [6]. Apical granuloma comprised 3.7% of all lesions, which was lower than the results of Bhaskar (12.0%) [10] and Tay (8.8%) [11]. Most apical granulomas in the current cohort were located in the maxilla, but no specific location has been reported in other studies [5-11]. Exostosis and dense fibrotic dental follicles were the two most common lesions in the others/miscellaneous group, which mostly occurred in the maxilla and were distributed in the sixth and second decades of life.

## Conclusion

The present study described in detail the frequency, age and sex distribution in a cohort of Taiwanese patients referred for histopathological examination. It showed trends similar to previous reports from other countries; however, some detailed information was different, perhaps due to the different criteria and different geographic distribution. Moreover, oral SCC and oral potentially malignant disorders comprised high percentages of all HN lesions in the cohort; nevertheless it should be cautioned that the sample being non-epidemiologic and would probably be greatly askew to a high rate of malignant/potentially malignant lesions when compared to an epidemiological sample.

## Abbreviations

HN: Head & neck; OMF: Oral and maxillofacial; SCC: Squamous cell carcinoma; HK: Hyperkeratosis; ED: Epithelial dysplasia; OSF: Oral submucous fibrosis; EH: Epithelial hyperplasia; ENT: Ear, nose & throat.

## Competing interests

The authors declare that they have no competing interests.

## Authors’ contributions

FL, KYH, and YKC are the primary writers of the manuscript and participated in the study implementing. YKC conceived of the study, and had made substantial contributions to conception and design, and revised the manuscript critically for important intellectual content. PHC implements all the required statistical analyses. WCW, LML, HCH, and JYC assisted in interpretation of data. CHC, and YKC are the principal investigators of clinical studies in this project. All authors read and approved the final manuscript.
